# Critical Airway Obstruction Caused by a Giant Multinodular Goiter: Successful Management With Emergency Thyroidectomy

**DOI:** 10.7759/cureus.90121

**Published:** 2025-08-14

**Authors:** Catalin D Cosma, Tudor Negruti, Ioana Cosma Epure, Calin Molnar, Dragos Molnar

**Affiliations:** 1 General Surgery, George Emil Palade University of Medicine, Pharmacy, Sciences, and Technology, Targu Mures, ROU; 2 General Surgery, Emergency County Hospital, Targu Mures, ROU; 3 General Surgery, George Emil Palade University of Medicine, Pharmacy, Sciences and Technology, Tîrgu-Mureș, ROU

**Keywords:** acute airway obstruction, emergency thyroidectomy, emergency tracheostomy, multinodular goiter, tracheomalacia

## Abstract

Acute airway obstruction secondary to a massive multinodular goiter represents a rare but critical surgical emergency, often occurring in patients with long-neglected thyroid disease. We report on an 85-year-old female who presented at Emergency County Hospital Tg Mures in March 2025 in critical respiratory distress, with documented tracheal deviation, orthopnea, and multiple episodes of cardiorespiratory arrest. Clinical and radiologic assessment revealed a giant anterior cervical mass with retrosternal extension and near-complete tracheal compression. Emergency airway control was achieved via awake fiberoptic intubation, followed by urgent total thyroidectomy. Intraoperative findings included significant tracheomalacia, prompting the need for concurrent tracheostomy. Postoperatively, the patient required intensive care monitoring and developed two systemic complications, a right hemispheric ischemic stroke and acute limb ischemia, both of which were managed conservatively. Histopathological analysis confirmed a benign multinodular goiter. This case emphasizes the importance of prompt recognition and surgical intervention in life-threatening compressive goiter, particularly in elderly patients, to prevent airway collapse and optimize outcomes.

## Introduction

Thyroid pathologies are usually managed electively, but in different circumstances, patients sometimes require immediate surgical intervention because of acute airway obstruction. The trachea can become compressed by massive thyroid enlargement, which occurs from benign goiter, hemorrhagic degeneration, or neoplasm. The condition requires immediate airway control and emergency thyroidectomy, as it leads to fatal respiratory distress. The emergency version of endocrine surgery, thyroidectomy, occurs infrequently because it presents as a rare and challenging procedure. Reports from Testini et al. indicate that, at a center level, emergency thyroidectomy occurs in less than 0.7% of all thyroid surgeries, demonstrating its rare occurrence yet essential need in critical situations [1]. The occurrence of goiter-related life-threatening airway compromise remains infrequent in developed countries because of early detection and treatment, while such cases can still occur due to delayed diagnosis or coexisting medical conditions or patient neglect among older adults [1,2]. Acute airway compromise occurs when thyroid enlargement leads to hemorrhage within nodules and tracheal deviation and compression.

Furthermore, neoplastic infiltration is to blame in the majority of cases. The clinical presentation of this condition includes hoarseness, together with orthopnea, stridor, and episodes of cardiorespiratory arrest. These patients require multidisciplinary coordination that centers on precise surgical techniques and close intensive care unit monitoring. The treatment of an elderly woman with critical airway obstruction from a giant multinodular goiter involved both tracheostomy and emergency total thyroidectomy. The case validates current research about emergency thyroidectomy indications, outcomes, and surgical challenges while demonstrating the need for urgent surgical intervention when compressive thyroid pathology causes acute respiratory failure.

## Case presentation

A female aged 85 presented to the emergency department after family members reported the sudden deterioration of her chronic respiratory symptoms, particularly severe dyspnea, orthopnea, and biphasic stridor. Her condition had rapidly deteriorated over the previous 48 hours. She had been aware of an expanding anterior neck mass that had been present for more than two decades. She had a progressively increasing visible deformity in the anterior neck, yet she had not consulted a doctor or sought any medical intervention. The patient developed respiratory difficulty, together with hoarseness, dysphagia, and anxiety attacks due to suffocation, and she could not sleep in the supine position in the last three weeks before hospital admission. No thyroid function tests or imaging studies, nor endocrine evaluations, were done in the last year.

Clinical examination revealed acute respiratory failure with 38 breaths per minute, while the peripheral oxygen saturation was at 84% on room air, but it improved to 90% with a non-rebreather mask. The patient had a heart rate of 118 beats per minute and blood pressure of 145/78 mmHg. The voluminous multinodular mass that was 15x16 cm in size was located in the anterior cervical region with a bosselated surface, firm consistency, and no skin changes. 

The mass extended into the suprasternal region and displaced the trachea visibly to the left. There was no tenderness or local warmth, and the mass elevated with swallowing. Bilateral carotid pulsations were reduced, and venous stasis was observed across the upper chest and neck, suggestive of local vascular compression. She could not lie in a horizontal position because it made her dyspnea worse. During the emergency evaluation period, she experienced two cardiorespiratory arrests, which were treated with immediate cardiopulmonary resuscitation, endotracheal suctioning, and pharmacological stabilization.

**Figure 1:**
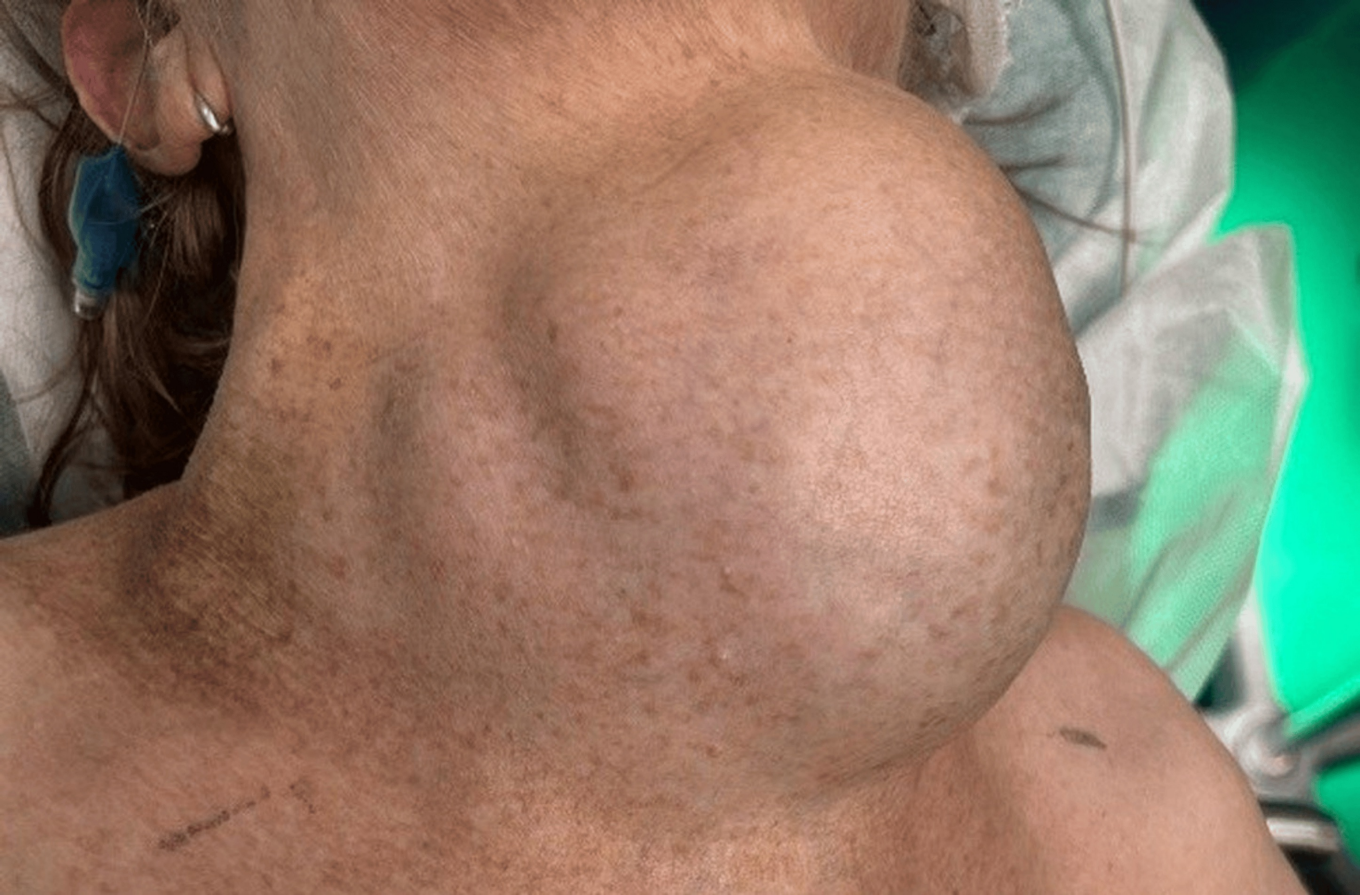
Preoperative clinical aspect of the neck

The image shows the anterior cervical region of the patient with a massive, multinodular goiter producing visible deformity, significant tracheal deviation, and prominent skin stretching. The mass extends below the sternal notch and is responsible for the patient's severe airway obstruction and stridor (Figure 1).

**Figure 2:**
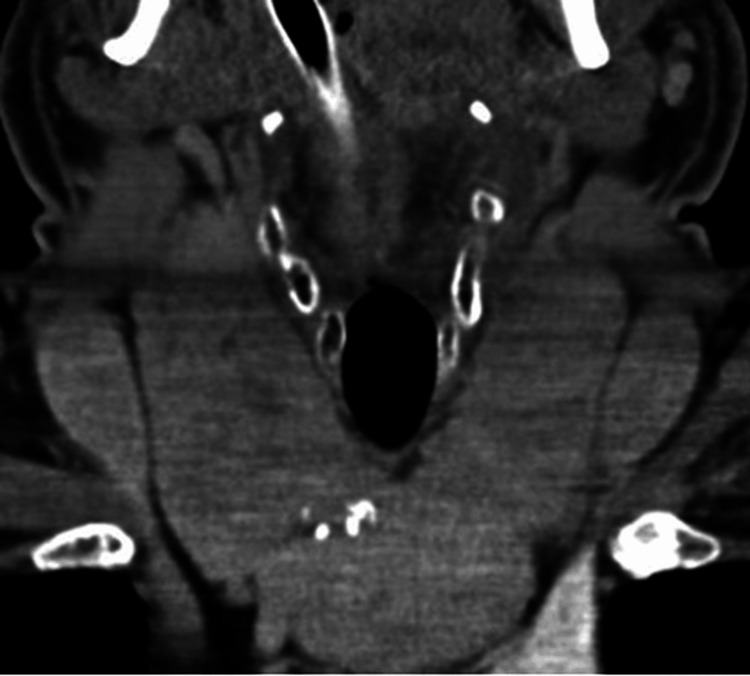
Preoperative contrast CT scan

The multidisciplinary team decided to perform emergency surgical decompression of the airway because of the life-threatening nature of the obstruction. The patient was taken to the operating theater with continuous monitoring. The preoperative assessment of the airway revealed that the tracheal deviation and anterior neck mass made the intubation process difficult. A successful fiberoptic intubation was performed under local anesthesia and conscious sedation. Once airway control was established, general anesthesia was induced with the use of short-acting agents. A pre-intubation contrast-enhanced CT scan revealed a giant substernal multinodular goiter with critical tracheal deviation and compression; the tracheal lumen was narrowed to a transverse diameter of less than 5 mm, and there was evidence of posterior displacement of vascular structures (Figure 2).

**Figure 3:**
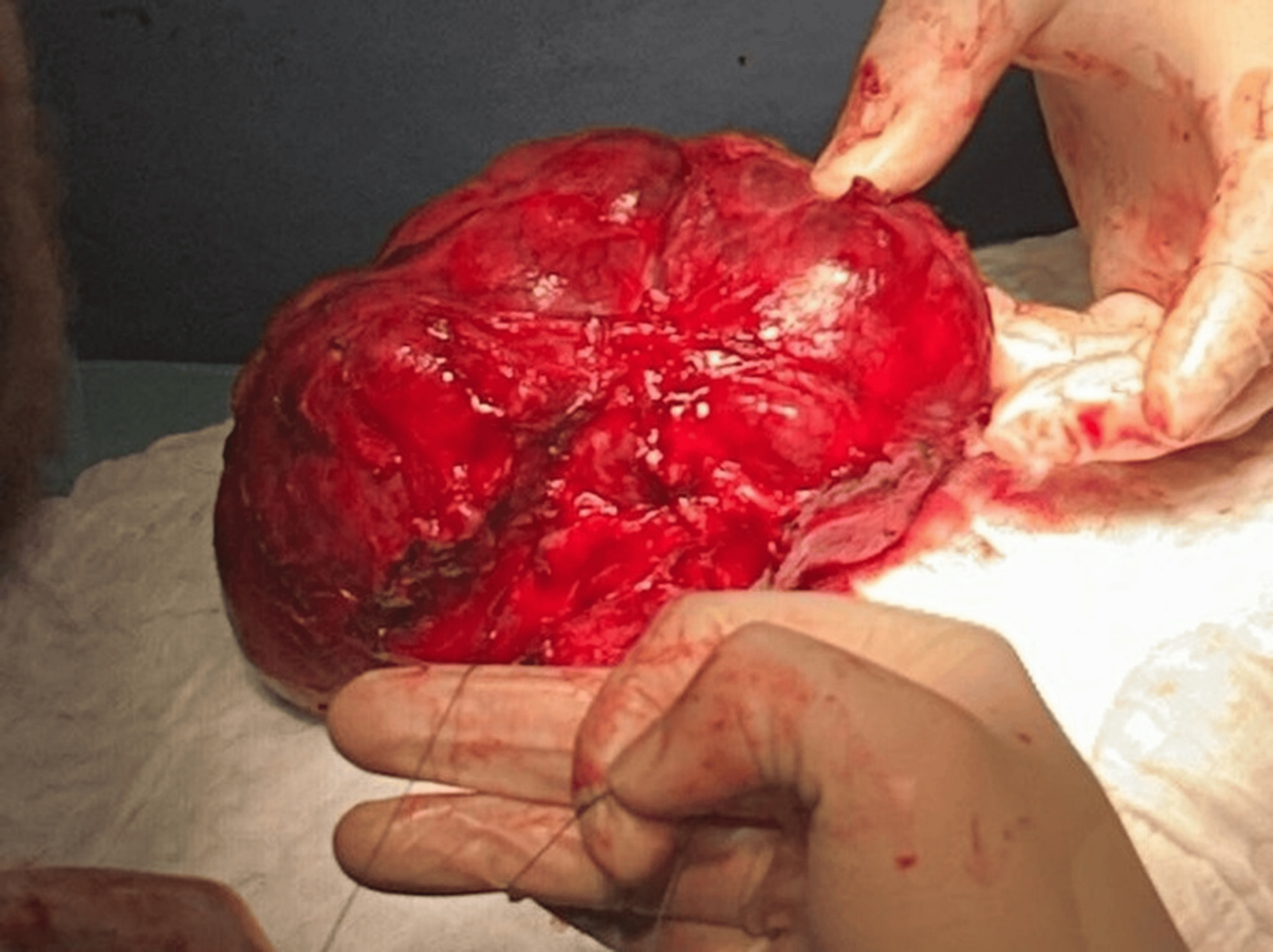
Intraoperative image of the excised goiter

The gross surgical specimen demonstrates a large, encapsulated multinodular thyroid gland with lobulated contours and visible areas of vascular congestion. The mass was delivered via cervical access and measured approximately 15 cm in its greatest dimension. This goiter caused critical tracheal compression and was removed as a monobloc during emergency total thyroidectomy (Figure 3).

Surgical access was gained through a transverse low-collar incision. Intraoperatively, the thyroid gland was found to be markedly enlarged, with both lobes extending retrosternally. Dissection was rendered difficult by dense adhesions to the strap muscles, trachea, and carotid sheaths. The isthmus was thickened and fibrotic. No suspicious lymphadenopathy or macroscopic evidence of malignancy was observed. The recurrent laryngeal nerves and parathyroid glands were carefully identified and preserved. Following subtotal mobilization, the goiter was resected in a monobloc fashion. The trachea was soft after decompression and lacked cartilaginous rigidity, as it had a visible pulsatile collapse during ventilation. Significant tracheomalacia was diagnosed, with a >50% reduction in tracheal lumen cross-sectional area during the intubation procedure. A surgical tracheostomy at the second tracheal ring was performed at the end of the procedure due to the high risk of airway collapse post-extubation.

The patient was transferred to the intensive care unit for postoperative monitoring and ventilatory support via tracheostomy. The tracheostomy tube was used for extubation the next day, and the patient was gradually weaned to room air over 72 hours. Postoperative laboratory studies revealed normal serum calcium and parathyroid hormone levels. Histopathological examination of the surgical specimen showed the presence of a benign multinodular goiter with stromal hyalinization, fibrosis, and coarse calcifications, but no capsular invasion or malignancy.

The patient developed two major non-surgical complications while in the ICU. An ischemic transient cerebrovascular event on postoperative day four, diagnosed with a CT contrast scan, which produced left hemispheric symptoms and cranial imaging confirmation, and right lower limb ischemia, which developed from atherosclerotic disease but received conservative treatment with anticoagulation (aspirin 200 mg with clopidogrel 57 mg) and limb elevation. Both complications received successful treatment while working with the neurology and vascular surgery teams. The patient experienced a gradual improvement in neurological status while peripheral blood flow to her right lower limb was reevaluated with local Doppler echography. Vascular surgery evaluation recommends amputation due to peripheral acute vascular ischemia that developed. The family and patient did not consent to the surgical intervention, and sepsis developed, resulting in the death of the patient.

## Discussion

Emergency thyroidectomy, although rare, is an essential procedure in cases of life-threatening airway obstruction due to thyroid disease. Goiters of significant volume, particularly those with retrosternal extension, can compromise the airway and necessitate urgent surgical decompression. Our patient presented with acute tracheal compression caused by a long-standing multinodular goiter, demonstrating stridor, orthopnea, and multiple cardiorespiratory arrests, which underscored the critical nature of the situation.

Emergency thyroidectomies constitute less than 1% of all thyroid operations, yet they are associated with significantly higher morbidity and mortality compared to elective procedures [1-3]. In a comprehensive literature review, Testini et al. emphasized the clinical value of emergency thyroidectomy in such life-threatening cases [1]. Gauger et al. described a broad range of acute thyroid presentations, including massive goiters and rapidly expanding malignancies such as thyroid lymphoma and anaplastic carcinoma [2]. Rohana and Hisham reported that acute airway compromise was the leading indication for emergency thyroidectomy in their experience [4].

In our patient, the obstruction was due to a benign but massive multinodular goiter with retrosternal extension. Although multinodular goiters generally develop slowly, sudden deterioration can occur due to spontaneous hemorrhage, inflammation, or positional tracheal collapse [4-6]. Imaging, especially contrast-enhanced computed tomography, is vital in assessing tracheal deviation, narrowing, and mediastinal extension, all of which help determine surgical urgency and approach [7,8].

A major intraoperative finding in this case was tracheomalacia. After the goiter was removed, the tracheal wall collapsed under positive pressure ventilation, reflecting chronic extrinsic compression of the tracheal cartilages over time. This is a well-documented complication, especially in elderly patients with long-standing goiters [4,9]. In such situations, extubation is unsafe, and an immediate surgical tracheostomy is often the only way to maintain airway patency [4,5,10].

Two unrelated complications developed postoperatively in our patient: an acute ischemic stroke and right lower limb ischemia. The first war was resolved successfully with medication; unfortunately, the other conservative treatment could not be applied, and written consent for amputation was refused by the family of the patient. These events reflect the elevated systemic risk profile in elderly individuals undergoing emergency surgery. Systemic stress, dehydration, and comorbid vascular disease can significantly raise the risk of thrombotic events in this population [6,11].

The decision to proceed with total thyroidectomy, rather than a limited resection, was made due to severe anatomical distortion and the need for complete decompression. Literature supports total resection in emergency settings to prevent recurrence, persistent symptoms, or the need for reoperation [5,12,13]. Postoperative outcomes were favorable, with no surgical site infection, recurrent laryngeal nerve palsy, or hypocalcemia noted.

Histopathological analysis confirmed a benign multinodular goiter, which aligns with findings from most emergency thyroidectomy specimens, although the possibility of malignancy must always be considered [14-16]. In some series, a substantial proportion of patients present with malignancy, including aggressive subtypes like primary thyroid lymphoma or metastases from other primaries [17-19].

In cases where there is significant retrosternal or mediastinal extension, the surgical approach becomes more complex and may necessitate extracervical access or collaboration with cardiothoracic surgery teams [20]. These scenarios require multidisciplinary coordination, advanced preoperative imaging, and surgical planning in experienced centers.

This case underscores several key points: the importance of early referral and elective surgical treatment for large goiters, the need for rapid intervention in the setting of acute airway obstruction, and the requirement for intraoperative flexibility when facing tracheomalacia or distorted anatomy. Surgical teams must be prepared for airway emergencies and postoperative challenges, particularly in geriatric patients.

## Conclusions

Emergency thyroidectomy represents a rare but essential surgical procedure when a massive goiter causes acute airway obstruction. The case shows that elderly patients with multiple health conditions can achieve positive results through immediate surgical intervention combined with team-based medical treatment. Delayed treatment not only increases operative risk but also raises the likelihood of postoperative complications, making proactive assessment and referral critical components of patient safety and long-term prognosis. The prevention of life-threatening complications depends on both early detection and prompt, scheduled surgical procedures for large goiters.
